# Goal alignment and unintended consequences of accountable care: How the structure of Oregon’s Medicaid coordinated care model shapes health plan–clinic partnerships

**DOI:** 10.1017/cts.2025.26

**Published:** 2025-02-06

**Authors:** Erin S. Kenzie, Jean Campbell, Mellodie Seater, Maya A. Singh, Alissa Robbins, Melinda M. Davis

**Affiliations:** 1 Oregon Health & Science University - Portland State University School of Public Health, Portland, OR, USA; 2 Complex Systems Program, Portland State University, Portland, Oregon; 3 Oregon Rural Practice-based Research Network, Oregon Health & Science University, Portland, OR, USA; 4 Clark County Public Health, Vancouver, WA, USA; 5 School of Medicine, University of Pittsburgh School of Medicine, Pittsburgh, PA, USA; 6 Oregon Health Authority, Portland, OR, USA; 7 Department of Family Medicine, Oregon Health & Science University, Portland, OR, USA

**Keywords:** Coordinated care, causal-loop diagram, Medicaid, health plan–clinic partnerships, accountable care, system dynamics

## Abstract

**Introduction:**

Accountable care models for Medicaid reimbursement aim to improve care quality and reduce costs by linking payments to performance. Oregon’s coordinated care organizations (CCOs) assume financial responsibility for their members and are incentivized to help clinics improve performance on specific quality metrics. This study explores how Oregon’s CCO model influences partnerships between payers and primary care clinics, focusing on strategies used to enhance screening and treatment for unhealthy alcohol use (UAU).

**Methods:**

In this qualitative study, we conducted semi-structured interviews with informants from 12 of 13 Oregon CCOs active in 2019 and 2020. The interviews focused on payer–provider partnerships, specifically around UAU screening and treatment, which is a long-standing CCO metric. We used thematic analysis to identify key themes and causal-loop diagramming to uncover feedback dynamics and communicate key findings. Meadows’ leverage point framework was applied to categorize findings based on their potential to drive change.

**Results:**

CCO strategies to support clinics included building relationships, reporting on metric progress, providing EHR technical assistance, offering training, and implementing alternative payment methods. CCOs prioritized clinics with more members and those highly motivated. Our analysis showed that while the CCO model aligned goals between payers and clinics, it may perpetuate rural disparities by prioritizing larger, better-resourced clinics.

**Conclusions:**

Oregon’s CCO model fosters partnerships centered on quality metrics but may unintentionally reinforce rural disparities by incentivizing support for larger clinics. Applying the Meadows framework highlighted leverage points within these partnerships.

## Introduction

Following the 2010 passage of the Affordable Care Act (ACA), state Medicaid programs have increasingly shifted to reimbursement models centered on the multiple aims of improving patient satisfaction, improving health outcomes, reducing healthcare costs, and improving clinician and healthcare workforce experience [1,2]. Accountable care organizations (ACOs) are partnerships of payers, providers, and community organizations that work together toward these aims. One mechanism by which this coordination takes place is the establishment of quality incentive metrics to assess the care patients receive. Partnerships between payers and primary care clinics are often leveraged to meet these metric goals [1].

In 2012, Oregon launched regional coordinated care organizations (CCOs), a community-based ACO model for adults and children enrolled in Oregon Health Plan, the state’s Medicaid program [3]. The state is seen as a “leader in health transformation” due in part to its ambitious ACO infrastructure in which CCOs assume financial risk for their patients, manage all aspects of care, and are held accountable for the quality of care delivered through various state-defined quality measures [3–5]. Oregon utilizes quality incentive metrics, which have been shown to play an important role in performance improvement [6–9]. However, prior research has indicated some unintended consequences of this model [9,10]. For example, one study found that CCOs prioritized larger and better-resourced clinics when providing support for colorectal cancer screening, potentially exacerbating rural disparities [9]. Smaller clinics, in general, have less capacity to engage in quality improvement (QI) [11]. To investigate how the structure of Oregon’s CCO model shapes payer–provider partnerships, we conducted a qualitative study aligned with an ongoing research initiative focused on improving screening, brief intervention, and referral to treatment (SBIRT) of unhealthy alcohol use (UAU) in primary care. The SBIRT metric has existed since 2013, but rates remain below targets [12]. We use causal-loop diagramming, a systems science approach, to identify and analyze feedback structures characterizing Oregon’s CCO model in order to glean insights about how the structure of the model shapes partnerships.

## Materials and methods

### Study Setting

Established in 2002, the Oregon Rural Practice-based Research Network (ORPRN) is a network of over 360 primary care clinics dedicated to practice-based and community research [13]. To address health outcomes of Oregonians with lower incomes, ORPRN collaborates with CCOs to conduct research and provide technical assistance to primary care clinics. One such project funded by the Agency for Healthcare Research and Quality (AHRQ) is titled Partnerships to Enhance Alcohol Screening, Treatment, and Intervention (ANTECEDENT), which addresses SBIRT and medication-assisted treatment for alcohol use disorder (MAUD). ORPRN designed the study to align with the state SBIRT metric and the existing infrastructure of SBIRT Oregon [14]. Study activities were approved by the Oregon Health & Sciences University Institutional Review Board through an expedited review (STUDY00020592). Additional findings from this study are outlined in separate publications [15,16].

#### Oregon CCOs

In 2019, 15 CCOs served just under 1 million patients enrolled in Medicaid across Oregon [17]. CCOs are regionally based and include a mix of for-profit and not-for-profit organizations [3]. At the time of our interviews, the populations served by CCOs ranged in size from roughly 10,000 to over 300,000 Medicaid enrollees. As part of their contract with the Oregon Health Authority (OHA), CCOs work toward meeting several quality metrics each year. Nineteen metrics, including SBIRT, were specified for 2019 [18]. The reported SBIRT rates for 2019 varied considerably between organizations [18]. Table 1 describes the characteristics of the Oregon CCOs at the time of our interviews.

**Table 1.** Characteristics of Oregon coordinated care organizations in 2019

Organization name	# of Enrollees^a^	SBIRT Rate 1^b^	SBIRT Rate 2^c^
Allcare health plan	49,830	23.9	57.3
Cascade health alliance	18,384	35.6	93.2
Columbia pacific	25,383	78.2	13.1
Eastern Oregon	51,565	69.3	58.2
Health share of Oregon	318,822	65.7	25.7
Intercommunity health network	55,014	50.2	85.4
Jackson care connect	31,561	46.7	26.1
PacificSource-Central	48,717	56.6	11.0
PacificSource-Gorge	12,076	54.3	10.4
PrimaryHealth Josephine	10,389	47.0	24.2
Trillium	91,134	60.9	42.9
Umpqua health alliance	27,761	44.9	49.6
Willamette valley community health	101,968	73.9	28.0
Advanced health	20,064	67.1	1.3
Yamhill community care	24,502	80.5	12.6

Data Sources: Medicaid Management Information System (MMIS); Decision Support/Surveillance and Utilization Review System (DSSURS).

a The data above represent all persons enrolled in a CCO and receiving Medicaid (Physical Health benefits) as of September 15, 2019.

b The denominator is all patients aged 12 years and older before the beginning of the measurement period with at least one eligible encounter during the measurement period. The numerator is the number of patients who received an age-appropriate screening, using an SBIRT screening tool approved by OHA, during the measurement period AND had either a brief screen with a negative result or a full screen.

c The denominator is all the patients in Rate 1 denominator who had a positive full screen during the measurement period. The numerator is the number of patients who received a brief intervention, a referral to treatment, or both that is documented within 48 hours of the date of a positive full screen.

Oregon’s CCOs have supported efforts to shift from traditional fee-for-service payment models to alternative payment models (APMs), such as value-based payments that link reimbursements and clinical goals to financial incentives [3,18]. This shift is considered a strategy for reducing costs while improving patient outcomes [6,19]. Since the implementation of value-based payments, the state has incrementally increased requirements for CCOs to expand the adoption of APMs; however, the degree to which they are used can vary greatly between organizations.

#### SBIRT metric

Nineteen states have quality metrics on substance use disorders, and many are transitioning to SBIRT, which is an evidence-based measure designed for primary care settings [20]. The United States Preventive Services Task Force recommends that clinicians screen adults for UAU and provide brief interventions to individuals engaged in risky drinking [21–24]. While referral to treatment is not currently the national recommendation [25,26], it is identified as a qualifying intervention within Oregon’s CCO quality metric for SBIRT [12]. Treatment is broadly defined and refers to any inpatient or outpatient substance use treatment, including mental health services and medication-assisted treatment provided within or outside the primary care setting.

Despite clear evidence demonstrating the effectiveness of SBIRT over the past 50 years, it continues to be inadequately performed in primary care settings [27]. Evaluation of 2017 US Behavioral Risk Factor Surveillance System (BRFSS) data showed that only 37.8% of adults remembered being asked about binge drinking during their last check-up; of those who reported binge drinking behaviors, only 41.7% were advised about the harms of this behavior, and only 20.1% were advised to reduce or quit [28]. Similarly, MAUD is prescribed to fewer than 9% of patients likely to benefit from it [29]. The SBIRT metric has existed in Oregon since 2013, beginning as a claims-based incentive measure that included only billable services for full screening and brief intervention [30,31]. In 2019, the CCO SBIRT claims-based measure was replaced by a reporting-only EHR-based measure [12].

### Participant Selection and Recruitment

We contacted representatives of all 13 CCOs that were active in 2019 and continuing into 2020. Two of the 15 CCOs were discontinuing services during the time of these interviews. We contacted CCO staff who worked directly with clinics or oversaw the CCO quality metric programs. Individuals were identified based on publicly available information and ORPRN and OHA’s prior experience with the CCOs. CCO staff were invited to participate via email with additional follow-up by phone or email to schedule. Participant recruitment and data collection were conducted by three qualitative analysts, two of whom had prior experience with practice facilitation (JC, MD), one of which also served as the study principal investigator (MD).

### Data Collection

Interviews were conducted in person between December 2019 and February 2020 by three analysts using a semi-structured interview guide. Questions in the interview guide related to strategies used by the CCO to achieve quality incentive metrics broadly, as well as specific approaches for addressing the SBIRT metric and the barriers clinics face to successful implementation (full guide available in Supplementary file 1). Interviews lasted 30–60 min and were conducted at a location chosen by the participant, most often their place of employment. Interviews were audio-recorded and professionally transcribed with participant consent.

### Data Analysis

#### Qualitative analysis

Validated transcripts were uploaded to ATLAS.ti and analyzed by members of the research team (EK, JC, and MD) according to thematic analysis [32]. All interviews were independently coded by two analysts and reviewed for agreement. Codes were developed *a priori* based on study priorities, and additional codes were added emergently during analysis. All inconsistencies were discussed among the analytic team until consensus was achieved. Initial themes identified through a series of analytic discussions guided retrieval and analysis of code-specific queries. An initial summary of themes and related quotations was discussed with the ANTECEDENT study advisory board, which consisted of patient, clinic, and health system partners, and was used to focus our analysis. We further refined our themes through additional group discussions and reviews with the full author team.

#### Causal-loop diagramming

Our team used causal-loop diagramming to illustrate complex dynamics found in the qualitative results [33,34]. This method was chosen because it elucidates aspects of system structure. The diagrams were produced during the late stages of thematic analysis by two trained analysts (EK and MS). First, we reviewed emergent themes and identified the core goal-directed feedback structure of the CCO incentive system. Then, we developed a series of five diagrams illustrating how components of the themes mapped onto that core structure. The diagrams adhered to standard causal-loop notation [33] and were produced using Kumu visualization software [35]. A table identifying each feedback loop and supporting qualitative information was developed in Microsoft Word.

#### Meadows’ leverage point framework

**Figure 1.**
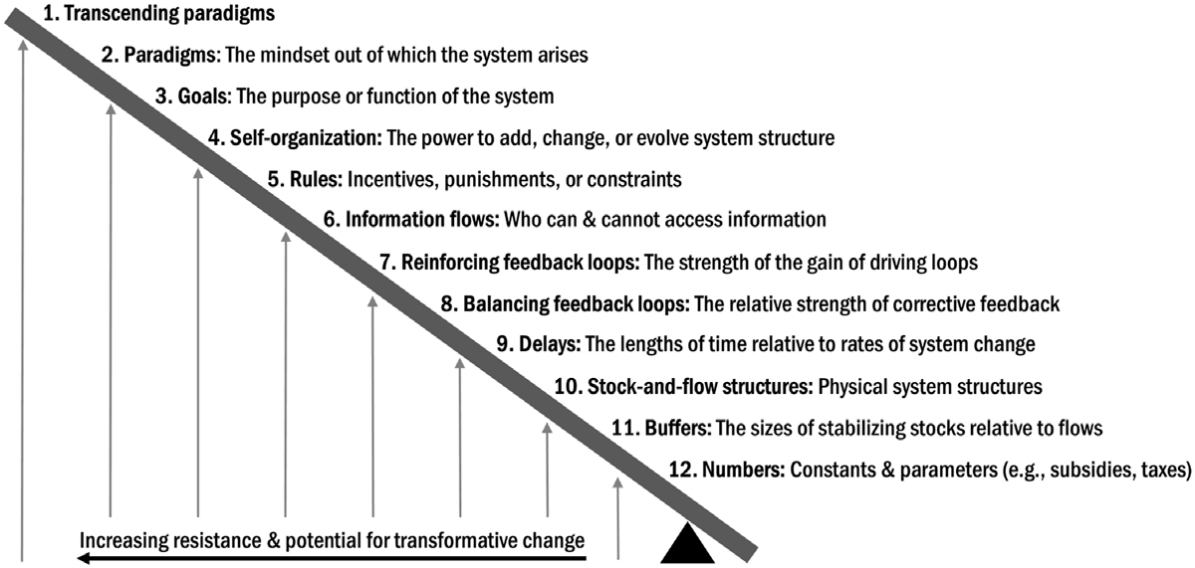
Meadows’ places to intervene in a system. Adapted from Meadows [36].

After completion of the causal-loop diagrams, our team applied a conceptual framework from Meadows [36] that ranks places to intervene in a system according to their capacity to affect change and their difficulty to implement, as shown in Figure 1. This framework is widely used in systems science to generate insight about potential interventions or to understand how aspects of system structure affect system behavior [37]. The same analysts (EK and MS) mapped our qualitative findings onto Meadows’ framework by reviewing the causal-loop diagrams and themes identified in qualitative analysis. The resulting table was reviewed by members of the study team to finalize the leverage points analysis.

## Results

A total of 23 individuals participated in 12 individual or small group interviews (1–4 CCO staff per interview). These participants represented 12 of the 13 CCOs active in 2019 and continuing into 2020. Interviewee roles included CCO leadership, QI specialists, direct clinic support staff, and analytic or reporting team members as summarized in Table 2.

**Table 2.** Coordinated care organization (CCO) key informant interview characteristics (*N*= 23)

Characteristic	Category	*N* (%)
Job type		
	Administrative	12 (52%)
	Direct support or QI specialist	9 (39%)
	Analytics or reporting	5 (21%)
Gender		
	Female	17 (74%)
	Male	6 (26%)
Years at CCO		
	<1 year	4 (17%)
	1–5 years	6 (26%)
	>5 years	4 (17%)
	Unknown	9 (39%)

### Payer-provided Support Strategies to Improve Clinic Performance and Achieve Quality Metrics

The CCOs” primary strategies for improving clinic performance included building and maintaining relationships, reporting on metric progress, technical assistance with EHRs, training and educational opportunities, cross-clinic and individual meetings, and APMs. This support was provided by CCO staff through in-person, virtual, and telephone interactions with clinic staff.

#### Building and maintaining relationships

Interviewees emphasized the importance of building and maintaining relationships between clinics and their CCOs in successfully achieving metric goals. Strong relationships allowed for open communication, ongoing dialog around improvement needs, and increased clinic buy-in and motivation for engagement, such as illustrated by the following CCO informant:

A lot of this really is relational work. So, maintaining good relationships with our partners is a really important part when it comes to thinking about change [. . .] So, if I were to maintain that great relationship, I [can] go into a clinic and provide coaching. We use PDSA [plan-do-study-act] cycles and more formal techniques when it comes to quality improvement, but it really does start with those relationships. (Participant 13, CCO 10)

CCOs often employ several staff to work directly with clinics. One participant (2, CCO 6) shared a strategy of delivering reports in person as a way to promote dialog, stating, “Some really good conversations come out of those [meetings]. We’re able to get questions in person that we might not get . . . if we just emailed.” According to informants, many of the CCO–provider relationships have been in place over long periods of time, which has allowed for deeper trust and collaboration to develop between all parties. CCO staff also discussed various changes occurring in their service areas and their need to prioritize establishing and building relationships to lay a foundation for success. These changes included shifting regional boundaries, changeover in staffing and leadership roles in CCOs, and clinics and shifts in OHA requirements. When relationships were lacking or fractured, interviewees highlighted it was imperative they be addressed.

#### Reporting and sharing metric progress

All CCO participants indicated that a major aspect of their role was to receive, validate, translate, and disseminate metric performance data to clinics. Dashboards and gap lists were the primary methods CCOs used to convey important, actionable performance information back to clinics. Dashboards provide comparative feedback on performance over time within and across CCOs. Gap lists are provided by CCOs to clinics to identify which patients have a gap in their care. These patients thus could be targeted for a screen or procedure that would improve their care and health outcomes, as well as improve the clinic’s metric performance. Providing actionable data and feedback to clinics and providers is imperative, as mentioned by one participant:

We have a fair amount of data analytics capability and quite a bit of data available to us here at the CCO. And so, we try to transform that into tools that are helpful for the clinics in doing their work. On the individual and clinical level, we’re working toward population health improvements at the CCO. But [then] we try to translate that into something that’s helpful for the clinic and for the staff and the clinician who are faced with this one patient today and what do they need. (Participant 10, CCO 3)

Metric data were shared with clinics in different ways, including individual communication and multi-clinic monthly meetings. One participant, for example, described monthly collaboratives with clinics to present data on metric performance and provide education around new metrics.

#### Electronic health record technical assistance

Participants noted that it was critical for clinical sites to accurately and consistently record information about clinical encounters in EHRs and to have an ability to pull that data to support metric reporting and enabling QI efforts. To address EHR challenges, CCOs engaged in strategies at the clinic level such as building EHR alerts that notify clinic staff when patients are eligible/due for certain screenings, writing or advising on queries to pull clinic reports, troubleshooting barriers in documentation workflow, identifying structured methods for data capture, adding new vocabulary terms to the EHR, creating order sets, and continuously educating sites about metric definitions. The following quote illustrates how CCOs provide EHR support to clinics to accurately capture the work clinicians are already doing:

A lot of what we do is helping providers get credit for work they’re already doing. I’m sure you’ve heard [that] providers are frustrated with healthcare and the measures. They want to doctor, they don’t want the documentation burden. They don’t want to click all the buttons. I think more and more they’re recognizing that [EHR documentation] the direction that things are going. But it’s challenging. (Participant 2, CCO 6)

The large number of EHRs used by clinics across the state present challenges for CCOs, as they must navigate the varying access to information and nuances that exist with each clinic’s system. One participant indicated that each of their clinics had a different EHR. Informants reported that larger, more commonly known EHRs are frequently associated with bigger clinics and health systems, while smaller, cloud-based EHRs are typically utilized by smaller, independent clinics. Larger EHRs were generally viewed more favorably by informants due in part to their ease of reporting. Participant 8 (CCO 11) shared, “We have found that so many of the smaller clinics, their EHRs just can’t produce (data) to the level needed . . . unless you have a report writer, it’s not feasible for a lot of the small clinics.” As such, obtaining reports can be a technical and financial burden for smaller clinics. The smaller clinics, however, were described as more “nimble” and able to respond more quickly. One participant described working with larger clinics to retrieve EHR data as “moving [a] monster” (Participant 8, CCO 11). Staff familiarity with EHR functionality and clinics’ perceived ability to change things within EHRs also varied, which complicated CCOs’ ability to provide technical assistance.

#### Trainings and educational opportunities

CCO trainings and other educational opportunities were considered by participants to be of considerable benefit to clinic partners in working toward improvement on incentive metrics and other community projects. Several CCOs described facilitating learning collaboratives as an arena to educate and discuss new metrics, review clinic-level data, and provide QI support. Educational trainings allowed CCOs to identify and respond to the most salient needs of clinics, as described by Participant 10 (CCO 3):

One of our strategies this year is providing broad quality improvement, education, and tools to everyone so that they can use this in pursuit of the CCO metrics, but also in pursuit of any other quality improvement projects that they may have at the time. So, we’re going to have some Lean [Six Sigma] training, and we asked them what they wanted to focus on to have kind of a coordinated community improvement project with some training and some tools. They’ve chosen initiation and engagement to alcohol and substance [mis]use treatment.

#### Alternative payment methodologies

Interviewees also described the CCOs’ strategies to incentivize improvements through APMs. Strategies included utilizing approaches designed to create a better understanding of the shift to value-based payments, aligning APMs with state performance measures, and seeking input from their member clinics regarding their priorities to better align payment structures with work the clinics are already invested in doing. Interviewees described the use of non-punitive APMs in incentivizing clinics to meet certain performance targets, using additional bonus payments for those that attain them. Clinics that meet more performance targets tend to receive greater APM reimbursements, often using this to re-invest in QI projects, specific care areas, partner organizations, and future pilots. As APMs become more widely adopted, CCOs have begun to link reimbursements to specific subpopulations.

Participants also described how APM structures can feel like they lack transparency or involve complicated formulas that reduce provider motivation for participating. They highlighted additional logistical and capacity-related barriers limiting participation in APMs and QI projects, especially among smaller clinics, with one participant stating, “[Smaller clinics] have not historically qualified for any of our participation in our [APM] or our quality pool payouts simply because they don’t have enough members assigned to them.” (Participant 19, CCO 4).

#### Causal structure of coordinated care model and support strategies

**Figure 2.**
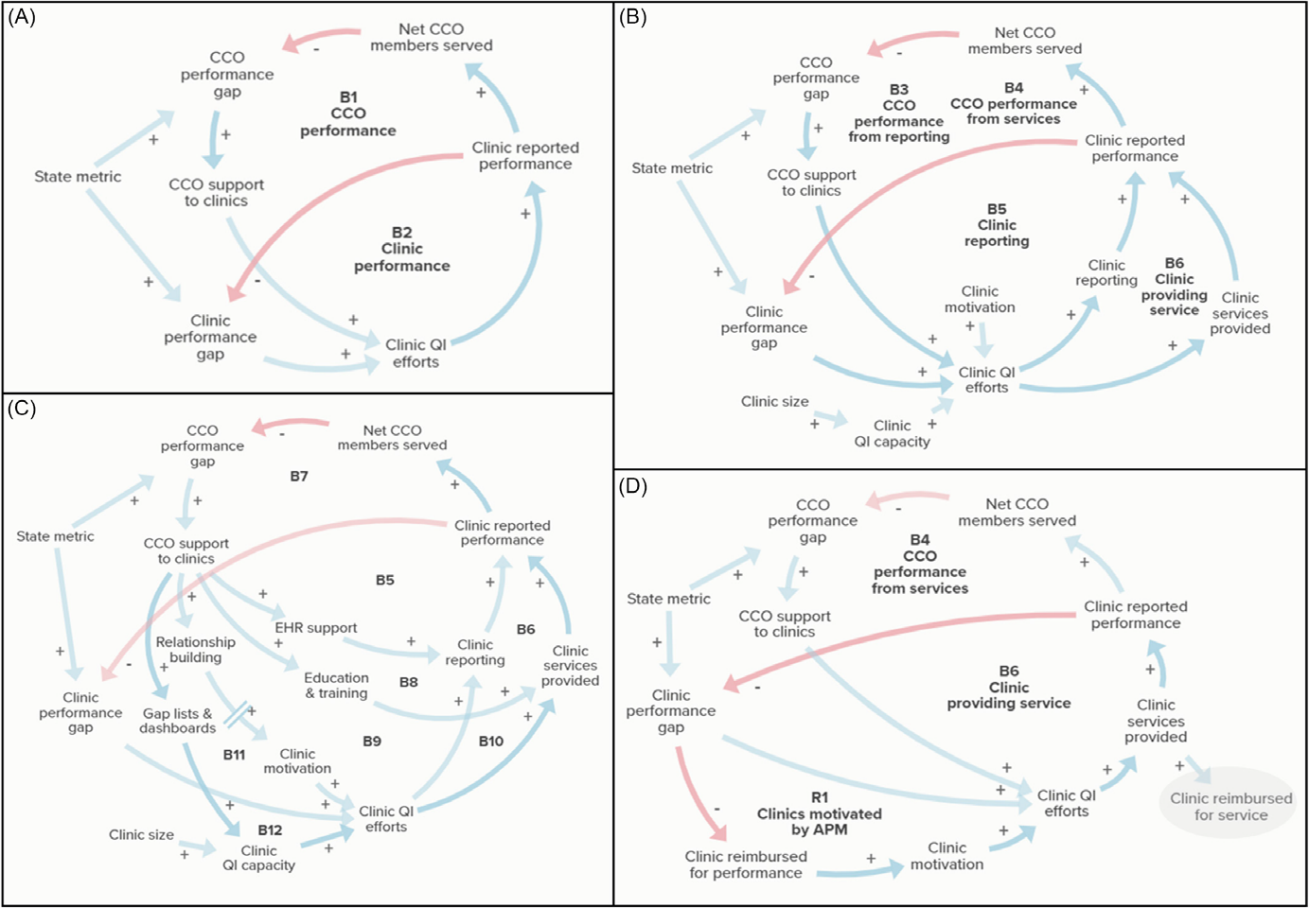
Causal-loop diagrams of coordinated care model and support strategies. Blue arrows with positive valence (+) indicate a change in the same direction (e.g., an increase in one variable leads to an increase in another). Red arrows with negative valence (−) indicate a change in the opposite direction (e.g., an increase in one variable results in a decrease in another). Dashed lines over the causal link between relationship building and clinic motivation indicate a time delay. Feedback loops are indicated with labels, with B indicating a balancing feedback loop and R indicating a reinforcing loop. Figure 2A describes the nested goal-directed feedback structure of the coordinated care model. Figure 2B provides additional detail about clinic QI. Figure 2C illustrates types of support coordinated care organizations (CCOs) provide to clinics found in our qualitative data. Figure 2D contrasts how clinics are reimbursed in the standard payment model (gray oval) with how reimbursement tied to performance in the APM strengthens the balancing feedback structure. Description and supporting quotations about individual feedback loops can be found in Supplementary file 2.

The series of causal-loop diagrams in Figure 2 illustrate the basic structure of the coordinated care model (2A), clinic-level factors influencing QI (2B), strategies used by CCOs to support clinic performance (2C), and the basic logic of APM (2D). The nested goal-directed balancing feedback loop structure in Figure 2A shows how CCO performance is dependent on clinic-reported performance, and both CCOs and clinics operate under benchmarks set by OHA. Figure 2B specifies that clinic-reported performance depends on clinics’ ability to provide the service and accurately report their metric performance. Clinics’ ability to engage in QI efforts is dependent on motivation and QI capacity, which is influenced in part by clinic size. Figure 2C illustrates how types of support provided by CCOs (EHR support, education and training, relationship building, and sharing data about metric progress through gap lists) support clinic improvement in different ways. Figure 2D describes how APMs are intended to strengthen clinic motivation by tying reimbursement to performance. Supplementary file 2 includes descriptions of each feedback loop in Figure 2 along with supporting quotations from our qualitative interviews.

### Prioritization of Clinics for Metric Support

**Figure 3.**
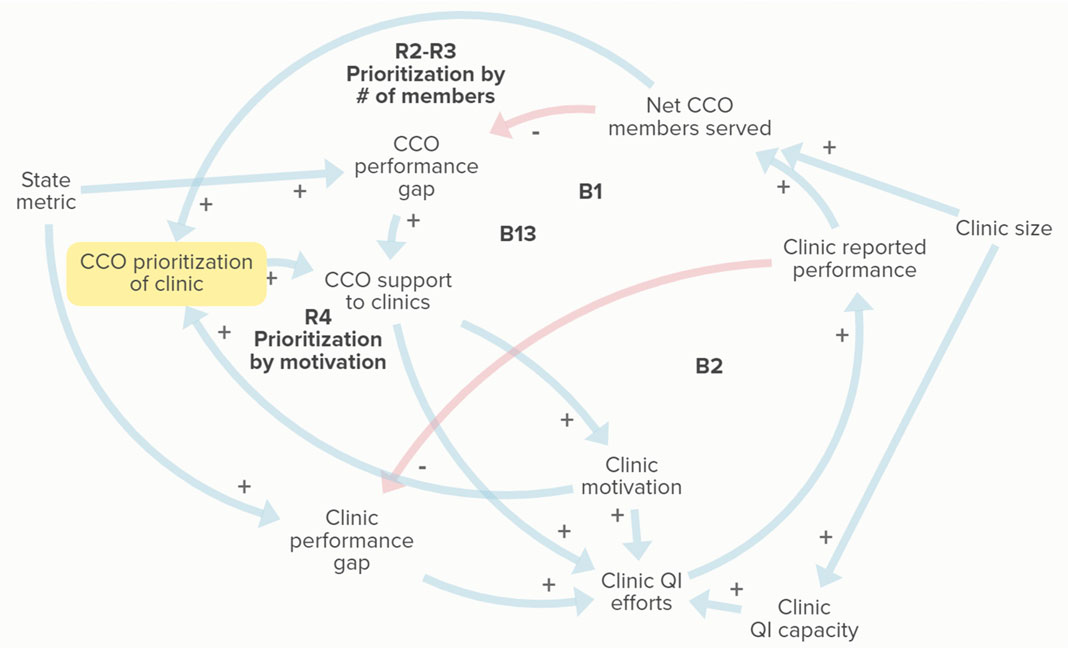
Causal-loop diagram of coordinated care organization (CCO) strategies for prioritizing clinics for metric support. Blue arrows with positive valence (+) indicate a change in the same direction (e.g., an increase in one variable leads to an increase in another). Red arrows with negative valence (−) indicate a change in the opposite direction (e.g., an increase in one variable results in a decrease in another). Feedback loops are indicated with labels, with B indicating a balancing feedback loop and R indicating a reinforcing loop. Loops R2–R3 indicate that clinics with high numbers of CCO members are prioritized for support. Loop R4 shows how highly motivated clinics can receive more support from CCOs and become further motivated. QI = quality improvement.

Figure 3 displays factors shaping how CCOs prioritize engagement with clinics for metric support. Many CCOs reported providing certain types of support, such as learning collaboratives or gap lists, to all of their clinics. Higher levels of support, however, were provided to clinics with more members. Participant 19 (CCO 4) shared, “In terms of prioritizing, it really is just those that hold the body of our members. Those 10 or so clinics have like 90% of our patient population, and so we focus our efforts there.” Informants reflected that clinics with a greater share of the CCO patient population tend to be larger clinics or those that are part of health or hospital systems, which often meant these clinic sites had their own QI teams and programs that were more developed (see loops R2–R3 in Figure 3). For simplicity, this is referred to as “clinic size” in our diagrams.

CCO staff also indicated that clinic engagement or interest is a factor in how they focus their efforts. One informant (Participant 13, CCO 10) likened the CCO–clinic relationship to one of a patient–provider relationship: “It’s kind of interesting because it’s in a way very similar to patient care. If you offer something and they don’t want it, then that’s okay. I’m here for you when you’re ready.” Another participant (10, CCO 3) spoke to this approach, stating, “We try to give everyone the same tools and supports. But the places where we’re able to refine those tools and kind of get more engagement is where we do spend more of our time. Because it’s hard to spend your time with someone that isn”t interested.” Loop R4 in Figure 3 shows how highly motivated clinics continue to get more attention due to their engagement.

Some CCOs also described approaching clinics based on metric performance, but strategies were mixed regarding prioritizing high or low performance. One CCO indicated that they targeted clinics that only needed small improvements to meet the benchmark, while another indicated that they approached clinics who are performing poorly on a certain metric.

### Application of Meadows’ Framework

As shown in Table 3, our qualitative findings about payer–primary care clinic partnerships under Oregon’s CCO model map onto multiple levels of Meadows’ framework. The basic structure of the CCO model (shown in Figure 2A) involves goal-directed feedback loops centered on *State metric* and relates to the goals level in Meadows’ framework, which has relatively high leverage. Strategies related to *Clinic motivation* relate to mindset/paradigms, which is also a potentially influential level. Social and cultural barriers related to UAU, including stigma, operate on the same level. Characteristics of the CCO model, such as levels of incentives, structures of metrics, and how CCO performance is defined, can be considered rules in Meadows’ framework. Most support strategies used by CCOs (*EHR support*, *Education & training*, and *Gap lists*) constitute improvements to information flows. Most strategies used by CCOs serve to strengthen reinforcing loops, while adopting APM introduces a reinforcing loop. Notably, our analysis showed that while barriers exist at the “lower” levels of Meadows’ framework, the CCO model did not rely on strategies at these levels.

**Table 3.** Application of Meadows’ framework

Place to intervene	Findings supportive of practice transformation	Barriers and unintended consequences
1. Transcending paradigms		
2. Paradigms	*Clinic motivation* influences *Clinic QI efforts*; CCOs support motivation through relationship building (Figure 2C) and APM (Figure 2D)	Social & cultural barriers such as stigma involve paradigms about UAU & substance use
3. Goals	Quality metrics set by OHA (*State metric*) are goals around which the CCO model is structured	Defining metric goals around *Net CCO members served* affects how CCOs prioritize clinics
4. Self-organization	CCOs have flexibility in their organizational structure and how they support clinics	CCOs’ ability to choose how they engage clinics may leave some higher-need clinics less supported
5. Rules (e.g., incentives, punishments, constraints)	Levels of incentives for CCO and clinic performance	SBIRT being a reporting-only metric instead of incentivized
6. Information flows	*EHR support* to improve *Clinic reporting*; *Education & training* to improve clinician skills; use of *Gap lists* and dashboards	EHR challenges, particularly for smaller clinics, impair clinic QI capacity and metric reporting
7. Reinforcing feedback loops	APM introduce a reinforcing feedback loop around clinic motivation (R1 loop in Figure 2); motivated clinics can be further motivated by CCO support (R4 loop in Figure 3)	Clinic prioritization strategies used by CCOs introduce reinforcing loops (R2–R4 loops in Figure 3) that work against goal-directed balancing feedback structure of CCO model and may perpetuate disparities
8. Balancing feedback loops	Core structure of CCO model is goal-directed balancing feedback (B1–B6 in Figure 2); types of CCO support strengthen these loops (B5–B12 in Figure 2).	
9. Delays		*Relationship building* takes time and benefits are not guaranteed
10. Stock-and-flow structures		
11. Buffers		Staffing time available for QI efforts is a limiting factor for CCOs and clinics
12. Numbers (e.g., subsidies, taxes, standards)		

*Note*: Model variables from Figures 2–3 are in italic.

APM = alternative payment model; CCO = coordinated care organization; EHR = electronic health record; OHA = Oregon Health Authority; QI = quality improvement; UAU = unhealthy alcohol use; SBIRT = screening, brief intervention, and referral to treatment.

## Discussion

Our study examined how Oregon’s coordinated care model shapes payer–primary care clinic partnerships generally and through a focused exploration of efforts to improve screening and treatment for UAU. Findings illustrate how payers can be active, influential actors in implementation – what Leeman and colleagues [38] term *support system actors*. CCOs supported clinics through building and maintaining relationships with clinics, reporting and sharing metric progress, EHR technical assistance, trainings, educational opportunities, and alternative payment methodologies. CCOs reported prioritizing larger clinics with more of their members and clinics that were highly interested in improvement. The causal-loop analysis identified the feedback structure underlying the CCO model, while the application of the Meadows framework facilitated the categorization of points of leverage.

### Goal Alignment of CCO Model Fosters Payer–Provider Partnerships

Our interviews showed that by aligning the goals of payers and clinics through incentive metrics, Oregon’s CCO model sets the stage for productive partnerships. Our causal-loop analysis indicates that the coordinated care model consists of two nested goal-directed balancing feedback loops in which CCO performance is dependent on clinic performance, and both CCOs and clinics operate under standards set by the state. In the systems science literature, goal-directed feedback loops are considered an engine of change in the sense that the tension created from the gap between current and desired performance leads to corrective action [39]. By incentivizing the CCOs to support clinic QI efforts, the coordinated care model is structured to improve clinic QI capacity. Figure 2C shows that the primary types of support provided to clinics by CCOs act on each part of the clinic performance balancing loop. In other words, the CCOs have developed ways of supporting clinics that relate to each opportunity for improvement.

Prior research supports the strategies used by CCOs in their work with clinics, such as building and maintaining relationships [9,40–42], trainings and educational opportunities, workforce development aimed at improving provider knowledge and confidence [43,44], value-based payments linked to quality goals [6,18,41], and provision of performance data (e.g., through gap lists and dashboards [9,45–47]. Many of the strategies for monitoring and supporting performance metrics mentioned by our interviewees align with those described by CCOs in a 2019 evaluation report, including regular internal monitoring, creating action plans, and implementing QI activities [48]. Our study aligns with prior research demonstrating the role of payer–provider partnerships in supporting clinic performance and quality of care [9]. Challenges reported by our interviewees reflect prior findings about the diversity of EHR types used by clinics [49,50] and difficulties customizing EHR tools to capture quality metric data [20,44,45,47].

### Clinic Prioritization Strategies May Perpetuate Disparities

CCOs reported prioritizing support to clinics that had a large proportion of CCO members and clinics that were highly motivated (shown in Figure 3). While these strategies may be a more efficient use of CCO staff time and result in quicker progress toward reaching metric benchmarks, they favor larger clinics with more existing QI infrastructure and available staff time. In other words, by optimizing around the thresholds of the quality metrics set by the state, CCOs may prioritize the “low-hanging fruit” of larger, better-resourced clinics at the expense of clinics with greater need. This pattern was seen across CCOs, including those that serve mostly rural areas, and is an unintended consequence of the CCO model also identified in prior work [9]. Engaging with a CCO takes staff time, so smaller clinics or clinics with fewer resources may not be able to utilize CCO support, even if motivated to improve performance. Because rural clinics are more likely to be smaller and have a lower capacity to engage in QI efforts [10,50,51], the current structure may unintentionally perpetuate health disparities in rural areas. These findings align with prior research suggesting that prioritizing larger clinics by CCOs may exacerbate rural health disparities [10,52].

Figure 3 shows how the reinforcing loops introduced by the CCO prioritization strategies work against the goal-directed balancing feedback structure of the CCO model. A restructuring of the CCO model to incentivize more equitable allocation of CCO support to clinics with greater needs would involve a critical look at the structure of the existing system. Defining CCO performance in terms of *Net CCO members served* (Figure 2), for example, encourages CCOs to focus on clinics with the greatest number of their members. A revised structure that considers clinic-level performance irrespective of number of members may enable small, rural clinics to receive more support from CCOs. To discourage a focus on minor improvements by clinics nearing the benchmark over clinics with more significant needs, the degree of improvement made by a clinic could be considered.

### Leverage Point Insights

Applying Meadows’ framework enabled us to identify the types of leverage implicit in various types of CCO support and barriers to change (see Figure 1 and Table 3). This analysis showed that Oregon’s coordinated care model utilizes multiple types of leverage, particularly those with medium or high potential for change. Barriers and unintended consequences existed across the spectrum of leverage points. None of our data mapped onto the highest leverage level in Meadows’ framework, transcending paradigms, which would entail a ground-up restructuring of the US healthcare system. This categorization of points of leverage used in CCO–clinic partnerships could inform future SBIRT implementation efforts or revisions to the Oregon CCO model by enabling implementers to target specific points of leverage, ensure a variety of kinds of leverage, or anticipating barriers or unintended consequences. While Meadows’ framework is typically used in a prospective way to identify potential points of leverage for planning future interventions, there is precedent for using it as an analytical tool [36]. Future research could specify processes for using this framework to analyze, improve, or compare existing programs or interventions.

### Limitations and future research

Our study has several limitations. First, our data pertain only to CCO staff perspectives. It is possible that clinic staff or providers may have a different experience of receiving support from CCOs. Second, the study did not assess other factors contributing to clinic metric performance or how CCO support affects patients. Future research could use mixed methods to examine the relationship between CCO support, clinic metric performance, and patient outcomes. Third, the study did not explore the impact of structural causes of substance use on patient outcomes under SBIRT, the patient experience as QI strategies were implemented, clinic access, or engagement of Medicaid health plan members and communities who have been disproportionately impacted by substance use. Future research should consider patient and community perspectives on SBIRT, other substance use interventions, like MAUD, and health equity through interviews or other avenues of participation. Fourth, our data are cross-sectional and largely represent the perspectives of CCO staff in late 2019. The introduction of CCO 2.0 and the start of the COVID-19 pandemic in the months following these interviews may have changed how CCOs approach their work with clinics, an angle that could be explored in future research. Fifth, data regarding the characteristics of clinics that take Medicaid insurance and therefore could be supported by CCOs (e.g., clinic size, rurality) were not available. Future research could quantitatively examine the relationships between clinic size, CCO support, and outcomes at patient, clinic, and CCO levels. Finally, this qualitative study does not constitute a comprehensive policy evaluation and potential changes to the metrics program should be thoroughly evaluated before implementation. Despite these limitations, this research identifies payer perspectives regarding partnerships with primary care clinics toward metric improvement, the basic causal structure of Oregon’s CCO model and identifies potential leverage points for future intervention.

## Conclusions

Oregon’s version of accountable care incentivizes payers to utilize a range of support strategies to improve clinic performance related to state-specified metrics. In our qualitative study, we found that CCOs varied in how they supported clinics and prioritized clinics to support. Using causal-loop diagramming, we identified the goal-directed balancing feedback structure of Oregon’s CCO model. Application of the Meadows framework allowed for categorization of points of leverage within health plan–clinic partnerships. Our findings align with concerns raised in prior research regarding potential exacerbation of rural disparities stemming from the way CCOs are incentivized by the state and suggest potential leverage points to facilitate clinic–CCOs partnerships and impact in future interventions.

## Supporting information

Kenzie et al. supplementary material 1 Kenzie et al. supplementary material

Kenzie et al. supplementary material 2 Kenzie et al. supplementary material

## Supplementary material

The supplementary material for this article can be found at https://doi.org/10.1017/cts.2025.26.
