# Supplementary material for: Goal alignment and unintended consequences of accountable care: How the structure of Oregon’s Medicaid coordinated care model shapes health plan–clinic partnerships
Source: J Clin Transl Sci. 2025 Feb 6;9(1):e46. doi: 10.1017/cts.2025.26 (PMC11975777; doi:10.1017/cts.2025.26)
Supplement: Kenzie et al. supplementary material 1 — Kenzie et al. supplementary material [file S2059866125000263sup001.docx]

**Supplementary file 1: Interview guide**

The questions below are the general topic areas we will explore with interview participants. These questions will be modified in light of what is learned during the interview and to fit the expertise of the interviewee.

Opening:

*Thank you for participating in this interview.*

*ANTECEDENT is designed to increase screening and brief intervention (SBI) and medication assistant treatment (MAT) for unhealthy alcohol use. We’ve partnered with the OHA Transformation Center and SBIRTOregon to align our efforts with the new CCO SBIRT incentive metric for the next two years.*

*You have been invited to be interviewed because of your role at the CCO. The purpose of this interview is to better understand the relationship with your member clinics and the extent to which you work with clinics to achieve the SBIRT quality incentive metric. We are also interested in understanding your approach to unhealthy alcohol use compared to unhealthy drug use (two different elements of the OHA SBIRT incentive metric). We anticipate our findings may inform other research and quality improvement initiatives – as well as be used to inform our current project which is called ANTECEDENT. Do you have any questions about the interview before we begin?*

*We’d like to record this conversation – we’ll get the recording transcribed and remove identifiers to facilitate our analysis process. Is it OK to turn the recorder on? [if yes, start audio recording].*

Semi-structured Interview Guide:

*First we’d like to start with some general questions about you and the CCO’s structure, then we’ll move on to focus more specifically on SBIRT.*

1. First, please tell us about yourself and your role in the CCO.

Probes:

- What is your background and training?
- How long have you been working for this organization, and what is your current role in the organization?
- What is your role with the CCO’s quality improvement and research initiatives?

2. Please tell us about how your CCO thinks about helping clinics change, specifically efforts you might have in place to support work related to the CCO incentive metrics?

Probes:

- General approach: outreach staff, in-clinic QI support, data relationships, advanced payment methodologies (APMS)
  - What partners (internally and externally) are at the table to support these initiatives?
  - When/how do you work with partners outside of your CCO to support work related to the CCO incentive metrics?
- When did you establish these structures and how have they changed over time?
  - How do you finance these structures?
  - What impact has CCO 2.0 had on your CCO’s quality improvement structure or priorities?

3.There are a number of performance metrics out there. How does your CCO decide what quality improvement initiatives or research projects to work on?

Probes:

- What process does your CCO use to make decisions about initiatives/projects?
- What factors shape whether or not the CCO decides to pursue or NOT pursue an initiative/project?
  - Intervention content (what you hope to implement, like SBIRT)
  - Process used to support implementation (how you’ll do it – QI support, funding, etc.)
  - Current context (organizational, policy)
  - Individual factors (like a clinic or provider says we want this)
- Can you describe an initiative/project your CCO recently decided to pursue – Why? What happened? How do you feel about this choice now?

4. How does your CCO decide which clinics to work with, for a specific quality improvement initiative?

Probes:

- How many clinics do you serve?
  - Is there a standardized process across clinics you work with, or do clinics opt to receive support? (if varied, can you give me an example of how?)
- What factors shape whether or not the CCO decides to approach a clinic?
- What factors shape whether or not the clinic decides to pursue an initiative/project with you?
  - Intervention content
  - Process used to support implementation
  - Current context (organizational, policy), individual factors
  - How much choice do clinics have in relation to what they participate in?
- Does/how does your CCO reimburse clinics for performance?
- Have you noticed anything about clinics that are or aren’t able to make improvements?

*Thanks for that general information about your CCO structure, priorities and processes. We’re now going to ask you a couple of questions about SBIRT and MAT as it relates to unhealthy alcohol and drug use.*

*We are interested in your perspective of similarities and challenges between different types of clinics – for example larger clinics (>10 primary care clinicians) compared to small to medium sized clinics (<10), independent versus affiliated with larger systems, rural versus urban, or others you’ve noticed.*

5. How has your CCO approached the SBIRT incentive metric, both in the past year – and in prior years if relevant?

Probes:

- What clinics have you worked with on SBIRT?
- What type of support has your CCO provided (expertise, financial, etc.)
- What types of challenges have you run into? What are you thinking of keeping or changing moving forward?
- What are the differences you see between different types of clinics (large vs small/medium practices, independent vs affiliated, etc.)?

6. How important is the SBIRT incentive metric to your CCO relative to the other OHA incentive metrics?

- Similar or different from prior years?

7. How have clinics responded to the requirement for screening for unhealthy alcohol or drug use? (“Unhealthy use” describes use of amount that can result in health consequences. Generally, any amount of certain drugs, 14 alcoholic drinks/week for men; 7/week for women)

Probes:

- What are the clinic’s perceptions of screening – is it seen as useful or not, how so?
- If it’s not perceived as useful, how does your CCO respond?
- Are perceptions of screening different for unhealthy alcohol versus drug use? Why?
- What are the differences you see between different types of clinics (large vs small/medium practices, independent vs affiliated, etc.)?

8. How have clinics responded to the requirement for brief intervention for unhealthy alcohol or drug use? (i.e. cognitive behavioral therapy, motivational interviewing)

Probe:

- What are the clinic’s perceptions of brief intervention – is it seen as useful?
- What are the differences you see between different types of clinics (large vs small/medium practices, independent vs affiliated, etc.)?

9. How have clinics responded to the requirement for interventions for patients with unhealthy alcohol or drug use – including Medication Assisted Treatment or Referral to Treatment? (Medication assisted treatment (MAT) is the use of medications in combination with counseling and behavioral therapies for the treatment of substance use disorders. The referral to treatment process consists of assisting a patient with accessing specialized treatment, selecting treatment facilities, and helping navigate any barriers)

Probes:

- What are the clinic’s perceptions of MAT – is it seen as useful? What are the challenges/facilitators for implementation?
- What are the perceptions of RT - is it seen as useful? What are the challenges/facilitators for implementation?
- Are there differences for MAT/RT for unhealthy alcohol use versus drug use?
- What are the differences you see between different types of clinics (large vs small/medium practices, independent vs affiliated, etc.)?

10. What support has your CCO provided to member clinics for SBIRT reporting?

Probes:

- How are clinics reporting SBIRT metrics? Are they required to report? What clinics are reporting? How often?
- What is the CCO’s role in SBIRT reporting? What support is the CCO providing?
- What are the challenges/opportunities here?
- In addition to CCO reporting, do clinics use their performance data internally, and if so how?
- If CCOs interpret data and share it back with clinics, how soon do they get feedback?

*ANTECEDENT is designed to address unhealthy alcohol use in primary care. ANTECEDENT is aligned with the Oregon Health Authority (OHA) Coordinated Care Organization (CCO) incentive metric for Screening, Brief Intervention, and Referral to Treatment (SBIRT) for unhealthy alcohol and drug use. The support provided in ANTECEDENT will be tailored to clinic, health system, and CCO needs. All participating clinics will receive Foundational Support–which includes assistance with SBIRT quality metric reporting and access to an intervention toolkit. Clinics will also be eligible for Supplemental Support–which includes 6-9 months of tailored practice facilitation support.*

11. What do you think are the key factors that would make SBIRT/MAT program implementation successful with your member clinics?

Probes:

- How would you structure the implementation process for this program?
- Are these factors present or absent?
- What could be done to [increase, decrease] these factors?
- Are there differences between different types of clinics (large vs small/medium practices, independent vs affiliated, etc.)?

12. What are some barriers or challenges to implementing a SBI/MAT program?

Probes:

- How might staffing, financial resources, or material resources (e.g., equipment, computers) impact your ability to implement this program?
- How confident are you that your member clinics could overcome these barriers or handle these challenges?
- How are these barriers or challenges addressed?
- Are there differences with large vs small/medium clinics

13. If you haven’t already, would you be willing to provide an introduction of this project to your member clinics? [explore strategies and timing]

- What clinics do you recommend
- When and who to approach

*Thank you for your time. This information will be used to inform refinement of interventions to increase implementation of unhealthy alcohol use interventions. We look forward to working with you and sharing our findings on this work.*
