# Supplementary material for: Goal alignment and unintended consequences of accountable care: How the structure of Oregon’s Medicaid coordinated care model shapes health plan–clinic partnerships
Source: J Clin Transl Sci. 2025 Feb 6;9(1):e46. doi: 10.1017/cts.2025.26 (PMC11975777; doi:10.1017/cts.2025.26)
Supplement: Kenzie et al. supplementary material 2 — Kenzie et al. supplementary material [file S2059866125000263sup002.docx]

**Supplementary file 2: Feedback loops and supporting documentation**

| **Feedback loop** | **Image & figure** | **Description** | **Supporting quotation(s)** |
| --- | --- | --- | --- |
| B1: CCO performance | 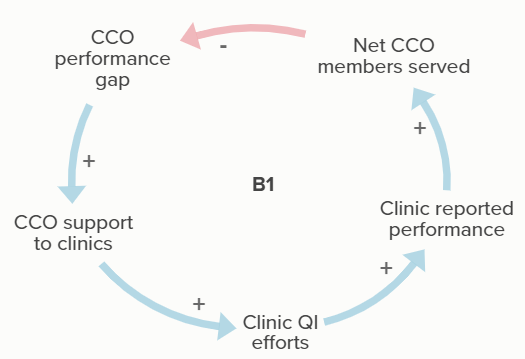  Figure 2A, 3 | As CCO performance gaps increase, the level of CCO support offered to clinics increases, which supports clinic QI efforts and leads to increased clinic reported performance. As the net number of members served increases, the CCO’s performance gap decreases. | “To the clinic and its staff, it's imperative to listen to what they're saying they need and then we work towards that direction. [It won’t work] if they don't have the capacity to assist us and to partner and be collaborative.” (Participant 11, CCO 3) |
| B2: Clinic performance | 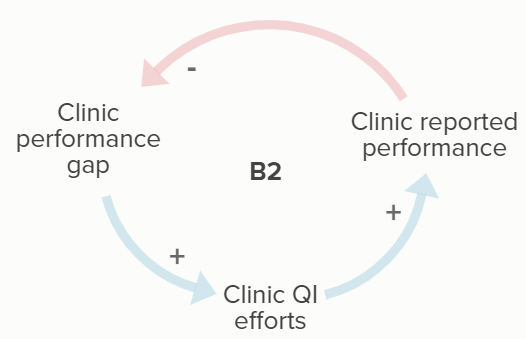  Figure 2A, 3 | When clinic performance is less than desired, clinics use QI efforts to improve their reported performance. | “I would say more often than not, they do want to participate. . . particularly because there are quality pool dollars that are attached to those metrics. So, if they see they're not doing well, oftentimes they will say yes. But if they say no, then that's okay. . .I just make myself available for them and say that when they're ready, they can come.” (Participant 13, CCO 10) |
| B3: CCO performance from reporting | 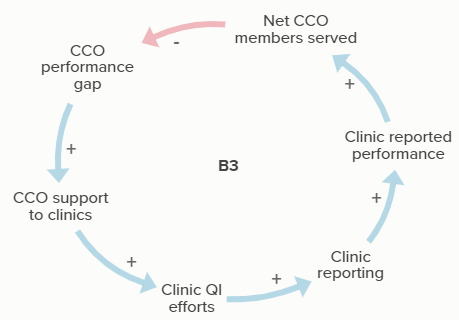  Figure 2B | When CCO performance on a metric is lower than desired, the CCO provides support to clinic QI efforts. Some of this support improves clinic reporting. When clinic reported performance increases, the CCO performance gap ultimately decreases. | “Yeah, and I think as a CCO, we're concerned about the data as well. Because if we don't have the data, we can't report and we don't meet our quality measures.” (Participant 10, CCO 3)  “And then, really, each year we had to take a look at what those measures are in order to determine where we need to focus our efforts. So, where do we think we're going to have a harder time reaching our target? Where do we feel pretty comfortable with current performance and we think that if we just let the clinics continue with the processes that they currently have, we should be fine.” (Participant 10, CCO 3) |
| B4: CCO performance from services | 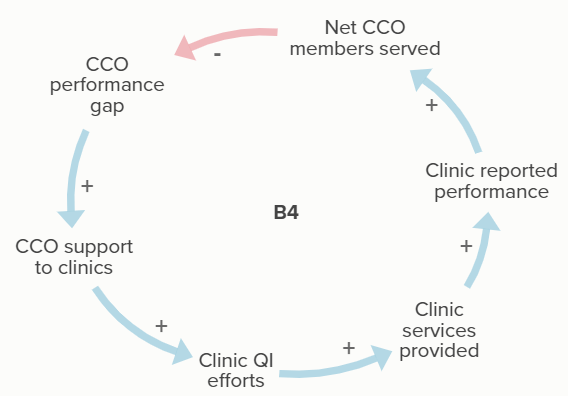  Figure 2B, 2D | When CCO performance on a metric is lower than desired, the CCO provides support to clinic QI efforts. Some of this support improves services provided by the clinic. When clinic reported performance increases, the CCO performance gap ultimately decreases. | “A lot of what we do is helping providers get credit for work they're already doing. . .But it's challenging. (Participant 2, CCO 6)  “As a clinic, the work that they do helps the CCO meet the metrics, or perform at a certain rate.” (Participant 8, CCO 11) |
| B5: Clinic reporting | 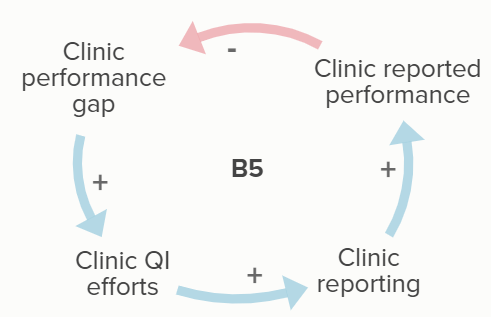  Figure 2B, 2C | When clinics’ performance on a certain metric is lower than desired, they engage in QI efforts to improve their ability to report on services provided. When their reported performance improves, their need to improve (*Clinic performance gap*) decreases. | “They want their performance rates to be reflected on the work that they know that they're doing. The staff are passionate about supporting their providers, the providers are looking at the numbers going, "What does this mean? I’m doing all of this." (Participant 11, CCO 3)  “One thing that I see from the clinics too, it's not just doing the work, it's doing the work within the parameters of the metrics that they get credit for too. So it's making sure that the procedure that they do falls under the right code or is coded the right way. It actually needs to show up in the systems too so that it actually falls into the metric.” (Participant 18, CCO 7) |
| B6: Clinic providing service | 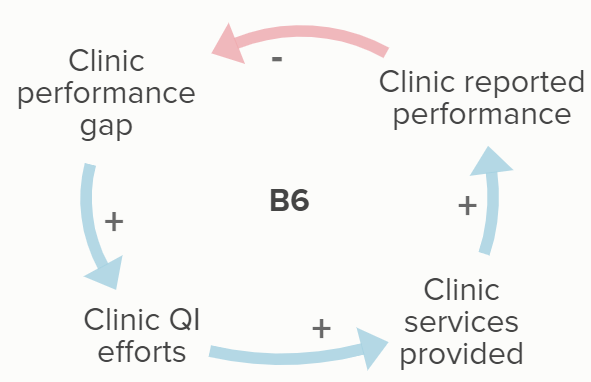  Figure 2B, 2C, 2D | When clinics’ performance on a certain metric is lower than desired, they engage in QI efforts to increase or improve the services provided. When their reported performance improves, their need to improve (*Clinic performance gap*) decreases. | “Not a lot of the providers or the staff have time to go in and try to figure out the why behind every metric. They're just doing the work at point of care. And so, they have to understand how is this meaningful at point of care? How can I actually make the changes at point of care? What is it I need to be doing?” (Participant 11, CCO 3)  “On the individual and clinical level, we're working toward population health improvements at the CCO. But [then] we try to translate that into something that's helpful for the clinic and for the staff and the clinician who are faced with this one patient today and what do they need.” (Participant 10, CCO 3) |
| B7: EHR support | 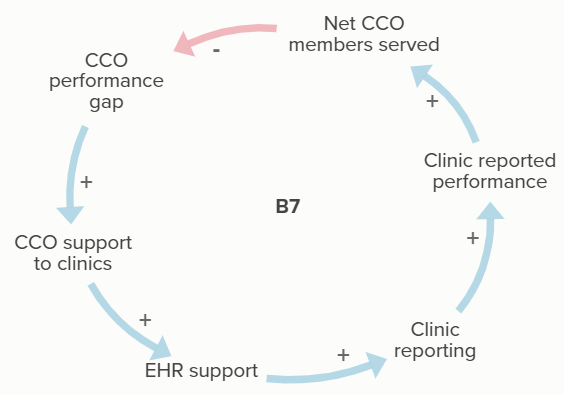  Figure 2C | EHR support provided by CCOs improves clinic reporting and the clinic’s reported performance. With greater performance more net CCO members are served, reducing the CCO performance gap and reducing the need for CCO support to clinics. | “We obviously have a lot of EHRs, so that makes it a little bit more challenging for us to provide technical assistance on reporting for the EHR based measures.” (Participant 6, CCO 9)  “Our EHRs in the area just don't capture [referrals to treatments]. And I think that's the same across the state. There just isn't a place to put it… So, it's really a CCO struggle to identify those non-structured data fields and provide them in a structured format, so.” (Participant 11, CCO 3)  “And so, we're going to work on how the providers use the EHR and make it easier and less time consuming for them and make sure we capture all of the information we need not just this SBIRT information and the depression screening information.” (Participant 10, CCO 3)  “Some clinics aren't able to report out of their EHRs, so while they would be able to participate when it was a claims based measure, now they're not able to participate in the incentive measure portion of it because they can't report out of their EHR or they don't have an EHR.” (Participant 10, CCO 3)  “We created note forms within the electronic health record that would help drive the conversation and document the steps that took place. Whether it's screening and results, created flow sheets to capture the results so then staff would enter all the findings from the laminated tools straight into the EHR. And then created a billing process.” (Participant 11, CCO 3) |
| B8: Education & training | 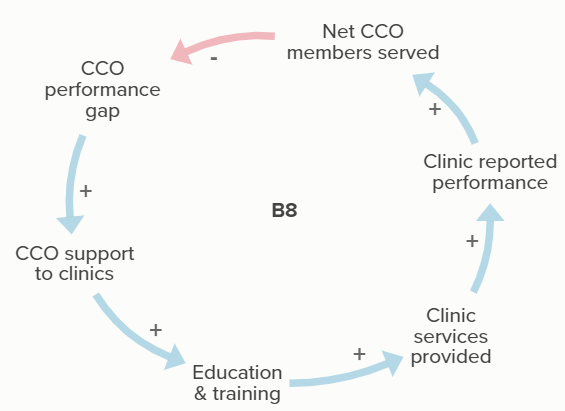  Figure 2C | Education and training provided by CCOs improves clinic reporting and the clinic’s reported performance. With greater performance more net CCO members are served, reducing the CCO performance gap and reducing the need for CCO support to clinics. | “One of our strategies this year is providing broad quality improvement, education, and tools to everyone so that they can use this in pursuit of the CCO metrics, but also in pursuit of any other quality improvement projects that they may have at the time.” (Participant 10, CCO 3)  “So we try, and do things to provide education around the measures, but not just the measures specifically, but the broader scope of the measure.” (Participant 2, CCO 6)  “We've also been explicit this year about trying to explain that we have different substance use disorder metrics, and why they relate or don't relate. . . And making sure that the clinics understand, clinically how that impacts work flow and then how that information gets extracted, and reported back.” (Participant 21, CCO 4)  “Now I feel we need to do a re-education of what addiction is and what addiction is not, in order to really meet the needs of our patients. And we needed to do it in a trauma informed way.” (Participant 9, CCO 12)  “So, we really approached it from let's start with the basic education and fundamentals. And then from the quality standpoint, once the training sessions were completed, we took the tools and we pretty much laminated them front to back. English on one side, Spanish on the other, and gave them out to all the providers with the how to interpret guide on a different sheet.” (Participant 11, CCO 3) |
| B9: Relationship building: reporting | 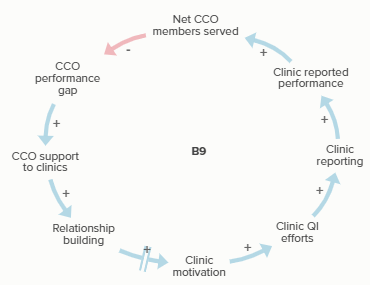  Figure 2C | Over time, relationship building efforts by CCOs support clinic motivation and subsequent clinic QI efforts. This increases clinic reporting and the clinic’s reported performance. With greater performance more net CCO members are served, reducing the CCO performance gap and reducing the need for CCO support to clinics. | “Our whole focus has been on establishing relationships with the providers and just patching over whatever rough spots there were before we got here.” (Participant 5, CCO 5)  “I think at a CCO level too, we really try to [troubleshoot] when providers contact us for data questions, answer the questions as comprehensive as possible. But also communicate that back to the quality team and then always try to support their internal quality teams as much as possible. That way we build that credibility. And make sure that we're supporting the internal side.” (Participant 11, CCO 3)  “And we work primarily with our clinics on those initiatives and on the incentive measures. We don't do a ton of direct member outreach, it's more relationship building with our clinics, then bringing data out to them about their specific claims based incentive measures and then working on workflow development and going over data trends with them.” (Participant 6, CCO 9) |
| B10: Relationship building: services provided | 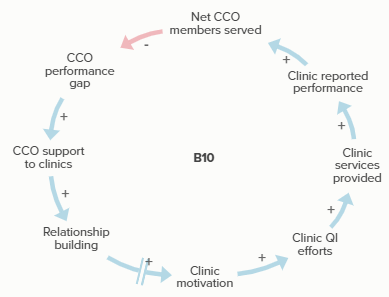  Figure 2C | Relationship building efforts by CCOs increase clinic motivation over time and support subsequent clinic QI efforts. This increases the clinic services provided and the clinic’s reported performance. With greater performance more net CCO members are served, reducing the CCO performance gap and reducing the need for CCO support to clinics. | “So, maintaining good relationships with our partners is a really important part when it comes to thinking about change [. . .] We think a lot about change management and about quality improvement. So, if I were to maintain that great relationship, I go into a clinic and provide coaching.” (Participant 13, CCO 10) |
| B11: Gap lists & dashboards: reporting | 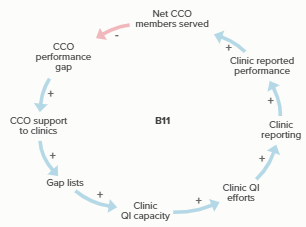  Figure 2C | Gap lists provided by CCOs increase clinic QI capacity and subsequent clinic QI efforts. This increases clinic reporting and the clinic’s reported performance. With greater performance more net CCO members are served, reducing the CCO performance gap and the need for CCO support to clinics. | “We have a fair amount of data analytics capability and quite a bit of data available to us here at the CCO. And so, we try to transform that into tools that are helpful for the clinics in doing their work.” (Participant 10, CCO 3)  “So with the metrics . . . the way the dashboards are designed is we know what our target is, we divide it out into quarters. And so we know that at the end of first quarter, this is where we should be at the end of second quarter, third and fourth quarter. And so the dashboards are either red, yellow, green or blue for being right on track. And so it's a really great visual right out of the gate for them to see that. “  (Participant 5, CCO 5)  “We have a dashboard that we've developed at the measure level that shows what the goal is, where we're at here today, where we’re at rolling 12, whether or not it's on track to meet the measure. We’ve got a color coding system. It's just yellow, red, green. Green's good, yellow is kind of between, red's not good so that helps us focus, and it's a good visual … to go through that and see where our weaknesses are and we'll focus more attention on those, unless we're just feel like we don't have a chance.” (Participant 1, CCO 6) |
| B12: Gap lists & dashboards: services provided | 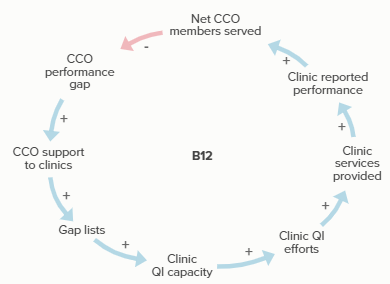  Figure 2C | Gap lists provided by CCOs increase clinic QI capacity and subsequent clinic QI efforts. This increases the clinic services provided and the clinic’s reported performance. With greater performance more net CCO members are served, reducing the CCO performance gap and the need for CCO support to clinics. | “Well, when I first started, we produced a gap list for the clinic, for each provider, that said, ‘These are the patients that essentially that qualify for this incentive measure and who haven't received a service yet. So, here's your list …’ It didn't really tell the providers how they were doing or what they needed to do with that, or set up a good process for how to do it… getting to something that's more actionable and then giving them tools to take action… It's that process there that drove the provider engagement and actually made incentive metrics in general feel very achievable.” (Participant 10, CCO 3)  “[E]ach of the organizations receive a performance dashboard. So how they are performing month over month, and closing gaps so it shows their denominator, or numerator, denominator performance rate, and then the CCO target. So they'll receive that, and then they also receive their gap list. So this is a list of members, and then there's different columns based by the metric, where that member has a gap in care. So if it's well-child checks, or adolescent well visits, or immunizations, or whatever the gap is, there's a notation on that document that helps the clinics hone in on which organization, or which members to reach out to. So it also includes demographics, so they can do phone calls, they can do mailers, whatever they need in order to reach out to members.” (Participant 8, CCO 11)  “Getting that data visualization tool now out to more than that one pilot group of providers… it puts little wins on their perspective that makes them feel like, achievable and that they're doing a good job. And they see and everybody understands what it all means.” (Participant 11, CCO 3) |
| B13: CCO performance due to clinic motivation | 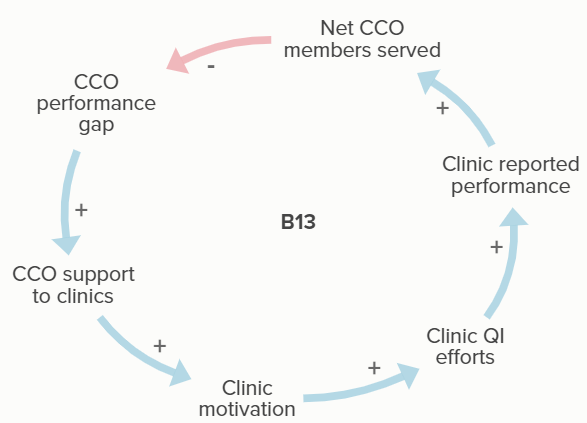  Figure 3 | As CCO performance gaps increase, the level of support offered to clinics increases. Clinic motivation increases with more support, improving clinic QI efforts and clinic reported performance. The greater the clinic performs, the greater net CCO members served, decreasing the CCO performance gap. | “The larger clinics with more resources definitely have an easier time, but I also think that it has to do with the providers’ buy-in and passion and knowledge about the subject.” (Participant 2, CCO 6)  “It's kind of interesting because it's in a way very similar to patient care. If you offer something and they don't want it, then that's okay. I'm here for you when you're ready.” (Participant 13, CCO 10) |
| R1: Clinics motivated by APM | 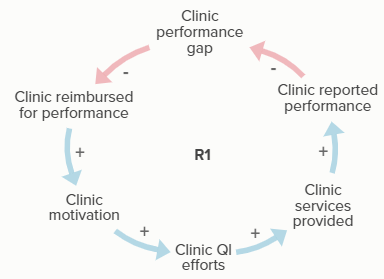  Figure 2D | Clinics are reimbursed for performance when their performance gap decreases. With more reimbursements, clinics are more motivated to participate in clinic QI efforts, which leads to increased services provided by the clinic, greater clinic reported performance, and more reimbursements for improved performance. | “We've tried to be creative in ways that we can seed and incentivize work that is really supportive of practice transformation and quality improvement. . . a big part of that is our alternative payment model.” (Participant 13, CCO 10)  “We try to incentivize groups based on their performance on the measures pretty directly tying the funding to their performance.” (Participant 12, CCO 2)  “It's been pretty critical for some of our clinics to get those bonus payments from the program and definitely they are able to use that money to put back into improving their quality programs.” (Participant 12, CCO 2)  “We actually have a very robust quality bonus payment that we give to our clinics on an annual basis. . . And then, we have a risk contract that our clinics, almost all of them participate in. And that allows them to receive funding if we are successful or not. . . it also allows them to receive funding for meeting the incentive measure. So they're not punished if they don't meet them, but the better they do, the more money that they get. . .those who aren't participating in our risk contract and aren’t eligible to receive funding for the incentive matters don't care about the incentive measures, and so they don't work on them.” (Participant 6, CCO 9)  “And I know that, one thing I think I can say really, I don't know, transparently, is that because we've used alternative payment methodology to drive priority, clinics are really concerned that they might do all of this work and that work won't be compensated. Because they don't have the full locus of control, right? They can do all the work, they can make those connections, but if there are other systemic issues or barriers on the behavioral health side, that their work will be, not in vain, but it won't be compensated.” (Participant 16, CCO 7) |
| R2-R3: Prioritization by # of members | 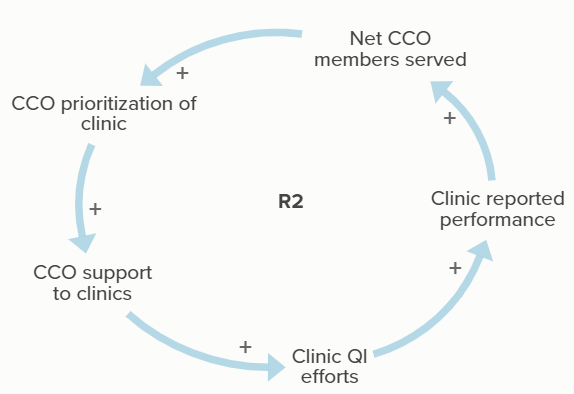  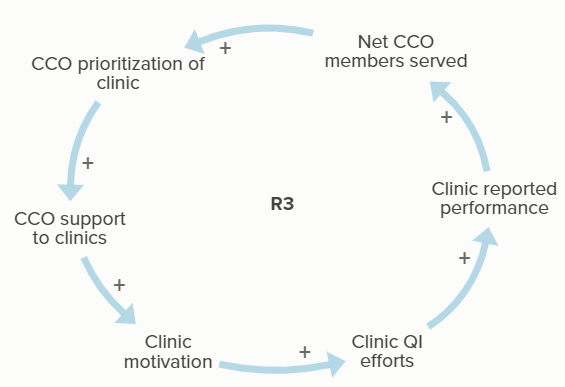Figure 3 | The greater a CCO prioritizes a clinic, the greater the level of support offered to that clinic. Clinic motivation and clinic QI efforts increase with more CCO support, improving the clinic reported performance. As clinic performance increases, more CCO members can be served, decreasing the need for the CCO to prioritize the clinic. | “Then looking at measures that will impact the greatest number of our membership—we look at all of those things and then determine which measures we'll focus on.” (Participant 2, CCO 6)  “In terms of prioritizing, it really is just those that hold the body of our members. Those 10 or so clinics have like 90% of our patient population, and so we focus our efforts there.” (Participant 19, CCO 4) |
| R4: Prioritization by motivation | 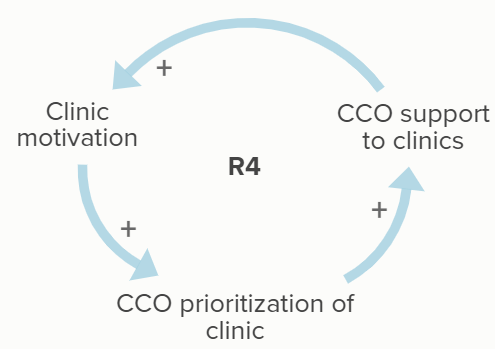  Figure 3 | CCOs prioritization of clinics increases the level of CCO support to clinics, which supports clinic motivation. As clinic motivation increases, the CCO’s prioritization of the clinic increases as well. | “We try to give everyone the same tools and supports. But the places where we're able to refine those tools and kind of get more engagement is where we do spend more of our time. Because it's hard to spend your time with someone that isn't interested.” (Participant 10, CCO 3)  “I look for who's already actively engaged because they're typically the ones volunteering to work on it. . . . We try to give everyone the same tools and supports. But the places where we're able to refine those tools and kind of get more engagement is where we do spend more of our time. Because it's hard to spend your time with someone that isn't interested. ” (Participant 11, CCO 3)  “I think one of the strategies is just engagement, and folks who are interested in engaging with us, and that can be part of it. So, we're looking for like where there's juice to be squeezed essentially and who would engage with us.” (Participant 13, CCO 10) |
